# Trends and hotspots in acupuncture treatment of rat models of stroke: a bibliometric analysis from 2004 to 2023

**DOI:** 10.3389/fnins.2024.1383283

**Published:** 2024-04-24

**Authors:** Song Li, Zhilin Huang, Tao Zhu, Anhong Dai, Xu Chen, Xiaolin Yang, Li Zhou, YiZhou Chen, Jing Shi

**Affiliations:** ^1^Second Clinical Medical College, Yunnan University of Traditional Chinese Medicine, Kunming, Yunnan, China; ^2^Department of Acupuncture and Moxibustion, First Affiliated Hospital of Yunnan University of Traditional Chinese Medicine, Kunming, Yunnan, China; ^3^Department of Acupuncture and Moxibustion, Yunnan Provincial Hospital of Traditional Chinese Medicine, Kunming, Yunnan, China; ^4^Department of Rehabilitation Medicine, Yan’an Hospital Affiliated To Kunming Medical University, Kunming, Yunnan, China

**Keywords:** acupuncture, bibliometric analysis, stroke, rat model, web of science

## Abstract

**Background:**

Acupuncture is a widely used clinical treatment method, and studies have confirmed its therapeutic effects on stroke patients. It can also reduce the burden on patients and society. Acupuncture treatment is a complementary and preventive treatment for stroke. However, there has yet to be a visual bibliometric analysis of the field of acupuncture for stroke rat models. This study explores future trends, research hotspots, and frontiers in acupuncture for stroke rat models over the past 20 years through investigation and visualization.

**Methods:**

We collected literature data on acupuncture treatment of stroke in rats from the Web of Science Core Collection (WOSCC) database from January 1, 2004, to December 31, 2023. Import into CiteSpace (version 6.2.R4) and RStudio for analysis by author, country/region, affiliation, annual publication, keywords, and journal visualization.

**Results:**

A total of 379 articles were retrieved, including articles from 16 countries, 258 research institutions, and 123 academic journals. The countries and institutions with the most publications were the People’s Republic of China (338) and the Fujian University of Traditional Chinese Medicine (43). Tao, Jing had the highest number of co-citations (144). The keywords and co-citation clustering show the main research directions in the field, including “artery occlusion,” “neural regeneration,” “stimulation,” “rapid tolerance,” “receptor,” “signaling pathway,” “apoptosis,” “oxidative stress,” “inflammatory response,” “endogenous neurogenesis,” “tolerance of local cerebral ischemic tissues,” “proliferation of reactive astrocytes” and “neuroprotective effect.” The intervention combines classical acupuncture treatment and modern technology (electricity) with electroacupuncture as a new intervention modality.

**Conclusion:**

This study demonstrates the increasing research on acupuncture for treating stroke in rat models. The country/region with the most publications is the People’s Republic of China. However, international cooperation still needs to be improved, and future researchers must strengthen international cooperation. In addition, in future studies, researchers should improve the overall quality of research results in this area and enhance research protocols.

## 1 Introduction

Stroke stands as the foremost contributor to adult mortality and disability globally (GBD 2016 Stroke Collaborators, 2019). According to the World Health Organization (WHO) surveyed in 2019, stroke is the second leading cause of mortality, accounting for 11% of deaths (Feigin et al., 2022). Despite the advancements in modern stroke treatment, a significant number of patients still endure long-term disabilities post-stroke, highlighting the pivotal role of rehabilitation in their recovery (Campbell and Khatri, 2020). Primary rehabilitation therapies include physical therapy, exercise and occupational therapy, compensatory training, motor imagery therapy, acupuncture, and massage (Zheng et al., 2018). However, enhancing the therapeutic effectiveness of current treatments and identifying additional therapies with alternative targets remain imperative. Acupuncture, originating in ancient China, has emerged as a complementary and alternative treatment method for stroke patients across various stages. Numerous studies have demonstrated the efficacy of acupuncture in the rehabilitation of post-stroke sequelae (Sun et al., 2023; Xiao et al., 2023), such as paralysis (Wang J. et al., 2020), aphasia (Wu et al., 2016), dysphagia (Xie et al., 2008), and cognitive impairment (Zhang S. H. et al., 2022).

Studies have confirmed the acupuncture’s mechanism of action in treating stroke patients. Clinical studies indicate that acupuncture treatment increases the fractional amplitude of low-frequency fluctuations (fALFF) values in regions including the cerebellum, anterior ventral gyrus, precentral gyrus, superior frontal gyrus, and parietal lobe, thereby enhancing regional brain functional activities and functional connectivity between cerebral hemispheres related to sensory integration, language processing, and motor coordination in stroke patients (Liu et al., 2021; Zhan et al., 2023). Moreover, acupuncture regulates neuroplasticity by stimulating neurogenesis, activating axon regeneration and sprouting, and improving the structure and function of the central nervous system in stroke patients, thereby reconstructing whole-brain function (Chavez et al., 2017; Qin et al., 2022). Acupuncture promotes improvement in movement, cognition, speech, and swallowing functions post-stroke, consequently enhancing the quality of life for stroke patients. In addition, acupuncture treatment could increase the blood supply to specific brain areas, promoting nerve function recovery, relieving muscle spasms, balancing muscle tension, and reducing inflammatory factor expression levels, thus promoting stroke patient recovery and symptom improvement (Qi et al., 2014; Zhou D. et al., 2022). After brain injury, the body will produce a series of pathological reactions, causing damage to brain tissue. By regulating this series of pathological changes, the degree of brain damage can be reduced. Multiple studies have confirmed that acupuncture can regulate oxidative stress (Han et al., 2018), inflammatory response (Li J. et al., 2021; Cai et al., 2022), cell apoptosis (Xing et al., 2021), neural regeneration(Zheng et al., 2016), Ferroptosis (Wu et al., 2023), microglia (Zou et al., 2022), mitochondrial autophagy (Wang et al., 2019) as well as glutamate, mRNA and other factors to alleviate Ischemic brain injury (Xing et al., 2018a,b). It is one of the therapeutic targets for stroke disease by regulating the pathological changes in brain cells post-stroke and promoting their compensation and recovery.

Bibliometric analysis emerges as a valuable tool for comprehensively assessing and quantifying research progress in a specific scientific field, allowing the mathematical and statistical measurement of the publication’s interrelationships and impacts. RStudio and CiteSpace software represent analysis tools combined with knowledge mapping widely used in information science, education, and medicine. At the same time, there have been bibliometric studies on acupuncture for stroke in recent years (Chun et al., 2023). However, currently, there is no relevant bibliometric analysis on acupuncture for stroke in a rat model. In this study, we employed RStudio and CiteSpace software to map studies and explore advanced and potential hotspots in acupuncture research for stroke rat models. Additionally, we extracted all the eligible literature from the Web of Science Core Collection (WoSCC) database from January 1, 2004, to December 31, 2023, for this study.

### 1.1 Data search

All data come from the Science Core Collection (WOSCC). The terms “acupuncture,” “stroke,” and “rat” were obtained from the MeSH.^1^ Researched papers published between January 1, 2004 and December 31, 2023 were collected and used: #1: ((((((((((TS = (acupuncture therapy)) OR TS = (acupuncture)) OR TS = (acupuncture point)) OR TS = (Acupuncture, Ear)) OR TS = (body acupuncture)) OR TS = (Auricular Acupuncture)) OR TS = (Electroacupuncture)) OR TS = (electroacupuncture)) OR TS = (Moxibustion)) OR TS = (needle)) OR TS = (scalp acupuncture). #2: ((((((((((((((((((((((((((TS = (Strokes)) OR TS = (Cerebrovascular Accidents)) OR TS = (Cerebrovascular Accidents)) OR TS = (CVAs (Cerebrovascular Accident)) OR TS = (CVAs (Cerebrovascular Accident)) OR TS = (Cerebrovascular Apoplexy)) OR TS = (Apoplexy, Cerebrovascular)) OR TS = (Apoplexy, Cerebrovascular)) OR TS = (Apoplexy, Cerebrovascular)) OR TS = (Apoplexy, Cerebrovascular)) Cerebrovascular)) OR TS = (Vascular Accident, Brain)) OR TS = (Brain Vascular Accident)) OR TS = (Brain Vascular Accidents)) OR TS = (Vascular Accidents, Brain)) OR TS = (Vascular Accidents, Brain)) OR TS = (Vascular Accidents, Brain)) OR TS = (Vascular Accidents, Brain)) Brain)) OR TS = (Cerebrovascular Stroke)) OR TS = (Cerebrovascular Strokes)) OR TS = (Stroke, Cerebrovascular)) OR TS = (Strokes, Cerebrovascular)) OR TS = (Strokes, Cerebrovascular)) OR TS = (Apoplexy)) OR TS = (Cerebral Stroke)) OR TS = (Cerebral Strokes)) OR TS = (Stroke, Cerebral)) OR TS = (Strokes, Cerebral)) OR TS = (Strokes, Cerebral)) OR TS = (Stroke, Acute)) OR TS = (Acute Strokes)) OR TS = (Strokes, Acute)) OR TS = (Cerebrovascular Accident, Acute)) OR TS = (Acute Cerebrovascular Accident)) OR TS = (Acute Cerebrovascular Accidents)) OR TS = (Cerebrovascular Accidents, Acute). #3: ((TS = (mouse)) OR TS = (mouse)) OR TS = (rat). We used #1 AND #2 AND #3 as search strategies in the WOSCC database. There were no language restrictions for all studies. We then converted the results into text files and further visualized and analyzed them.

### 1.2 Inclusion/exclusion criteria

**Figure 1:**
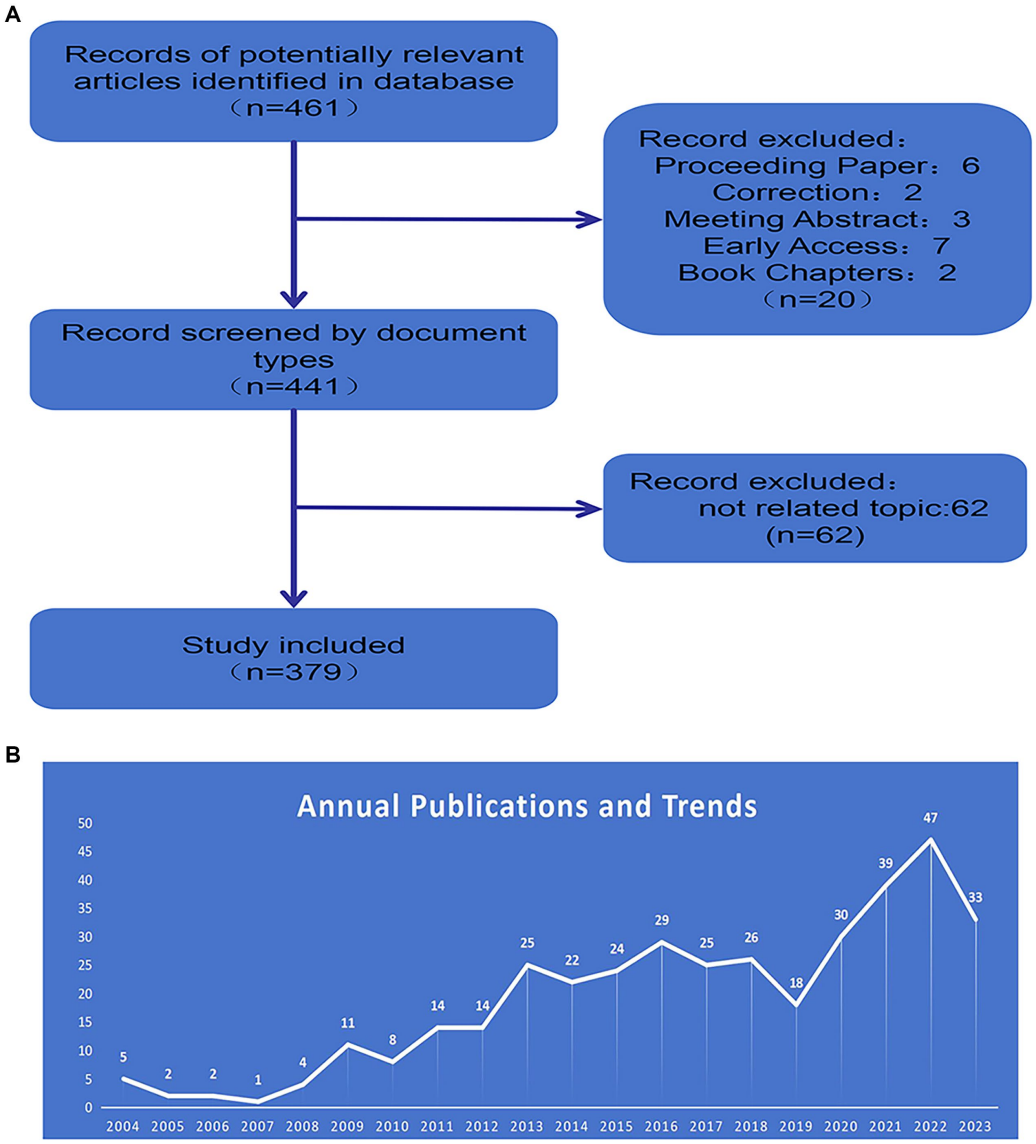
**(A)** Flow chart detailing the process of article selection. **(B)** Annual number of WoS publications in the field of acupuncture for stroke rat models.

After conducting our search on the Web of Science (WOS) database, 461 articles were identified using our predefined search formula. The inclusion criteria were: (1) Original articles; (2) Time frame: articles published between 2004 and 2023; (3) Studies on rat models of acupuncture for stroke; (4) Articles and Reviews. The exclusion criteria were: (1) studies on brain diseases other than stroke; (2) clinical studies; (3) early access, Proceeding Paper, Meeting Abstract, and Book chapters; and (4) retracted or overlapping studies. We screened 441 papers for the first time based on the inclusion and exclusion criteria. Two independent researchers screened the remaining documents by examining each title and abstract, excluding 62 papers that did not comply with the study content, ultimately including 379 records in the visual analysis (Figure 1A).

### 1.3 Data analysis

We used RStudio and CiteSpace (version 6.2.R4) software to screen and analyze publications based on year of publication, country, journal, research institution, author and keywords, visual classification, and analysis.

The different nodes in the graph indicate the different objects analyzed, and node diameter size suggests the occurrence frequency. Lines between nodes represent links, indicating the co-occurrence of these two nodes in the same study. The line’s color represents the time of the first co-occurrence, and the thicker line indicates a more vital link between the two (Li Y. X. et al., 2023).

The parameters of CiteSpace were such that for all calculations, from January 1, 2004, to December 31, 2023, the threshold for “first N% of each slice” was 50, and “each slice was 1 year.” We apply the log-likelihood ratio (LLR) algorithm to extract cluster labels.

## 2 Results

### 2.1 Published year

This study ultimately included 379 papers published in acupuncture for stroke rat models over the past 20 years, with the number of articles published per year and the trend shown in Figure 1B, from 5 papers published in 2004 to 33 papers published in 2023. Generally, research in acupuncture for stroke rat models shows an increasing trend, but there were minor fluctuations in this field in 2007 and 2019. The number of 47 papers published in 2022 is 9.4 times higher than in 2004, and there is a slight decrease in the number of papers published in 2023.

### 2.2 Countries/regional

**Figure 2:**
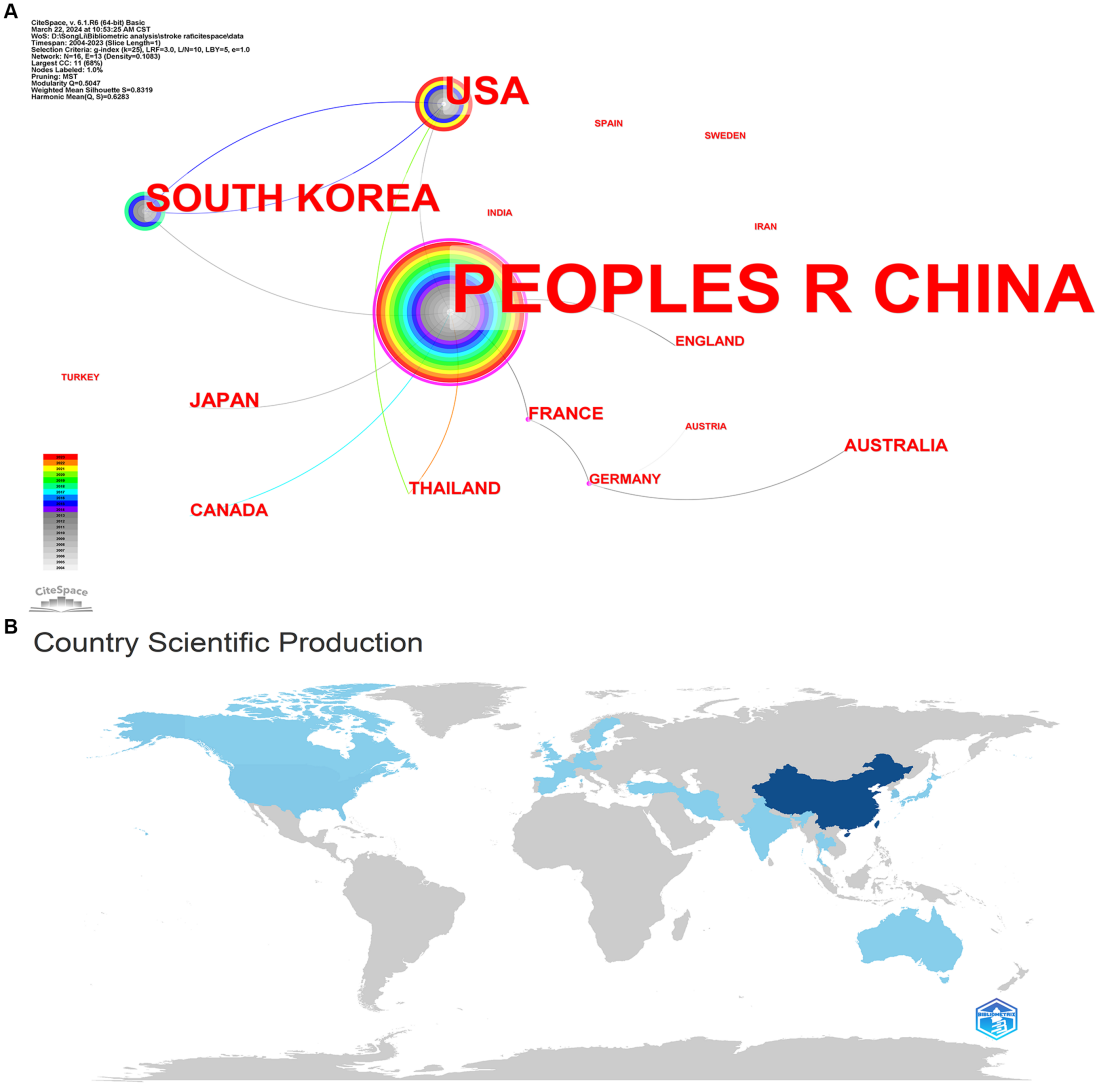
National distribution of published papers in the rat models of stroke treated with acupuncture **(A,B)**.

We used CiteSpace and RStudio to visualize and analyze the countries/regions for the papers on acupuncture for stroke rat models (Figures 2A,B). The People’s Republic of China published 338 papers, accounting for 89.18% of the 379 papers, with the highest number of published research papers. The USA followed this (6.33%), followed by South Korea (5.80%), Japan (1.06%), and AUSTRALIA (0.79) (Table 1). In terms of country cooperation, The People’s Republic of China was the country with the highest link centrality (0.70), followed by France (0.40), Germany (0.32), and the USA (0.1). The low link centrality of other countries reflects the relatively high level of cooperation between The People’s Republic of China and other countries. The visual international collaborative network shows that The People’s Republic of China has a high level of cooperation with the USA and South Korea. This result suggests that The People’s Republic of China, the USA, and South Korea are important in this research area. However, there still needs to be more international cooperation between countries.

**Table 1:** Top 5 countries/regions with the most publications on acupuncture for stroke rat model.

Rank	Countries/Regional	Counts	Centrality	%(of 379)
1	PEOPLES REPUBLIC OF CHINA	338	0.70	89.18
2	USA	24	0.10	6.33
3	SOUTH KOREA	22	0.00	5.80
4	JAPAN	4	0.00	1.06
5	AUSTRALIA	3	0.00	0.79

### 2.3 Journals analysis

We used RStudio software to analyze the journals that included 379 papers. A total of 123 journals were involved. Table 2 shows the top 10 journals by journal publication volume. Evidence-Based Complementary and Alternative Medicine published the most papers (37). Neural Regeneration Research published the second-highest number of documents (36). Acupuncture in medicine has the third largest published paper (18). It shows that Evidence-Based Complementary and Alternative Medicine journals are essential in this field. Regarding publication quality, the top 10 journals in terms of the number of publications had an impact factor of 2.5 to 6.1. Despite this, the top 10 journals with the most significant number of publications, Evidence-Based Complementary and Alternative Medicine and Alternative Medicine, two journals, were excluded from the Science Citation Index in 2023. indicating that the overall level of therapeutic publications could be higher and that high-quality research is still needed.

**Table 2:** The 10 journals with the highest frequency values in the rat models of stroke treated with acupuncture.

Ranking	Journal	Articles	IF(2023)
1	Evidence-Based Complementary and Alternative Medicine	37	0
2	Neural Regeneration Research	36	6.1
3	Acupuncture in Medicine	18	2.5
4	Brain Research	10	2.9
5	International Journal of Molecular Medicine	9	5.4
6	Scientific Reports	9	4.6
7	BMC Complementary and Alternative Medicine	8	0
8	Molecular Medicine Reports	8	3.4
9	Neuroscience Letters	8	2.5
10	PLOS One	8	3.7

### 2.4 Co-cited journals

**Figure 3:**
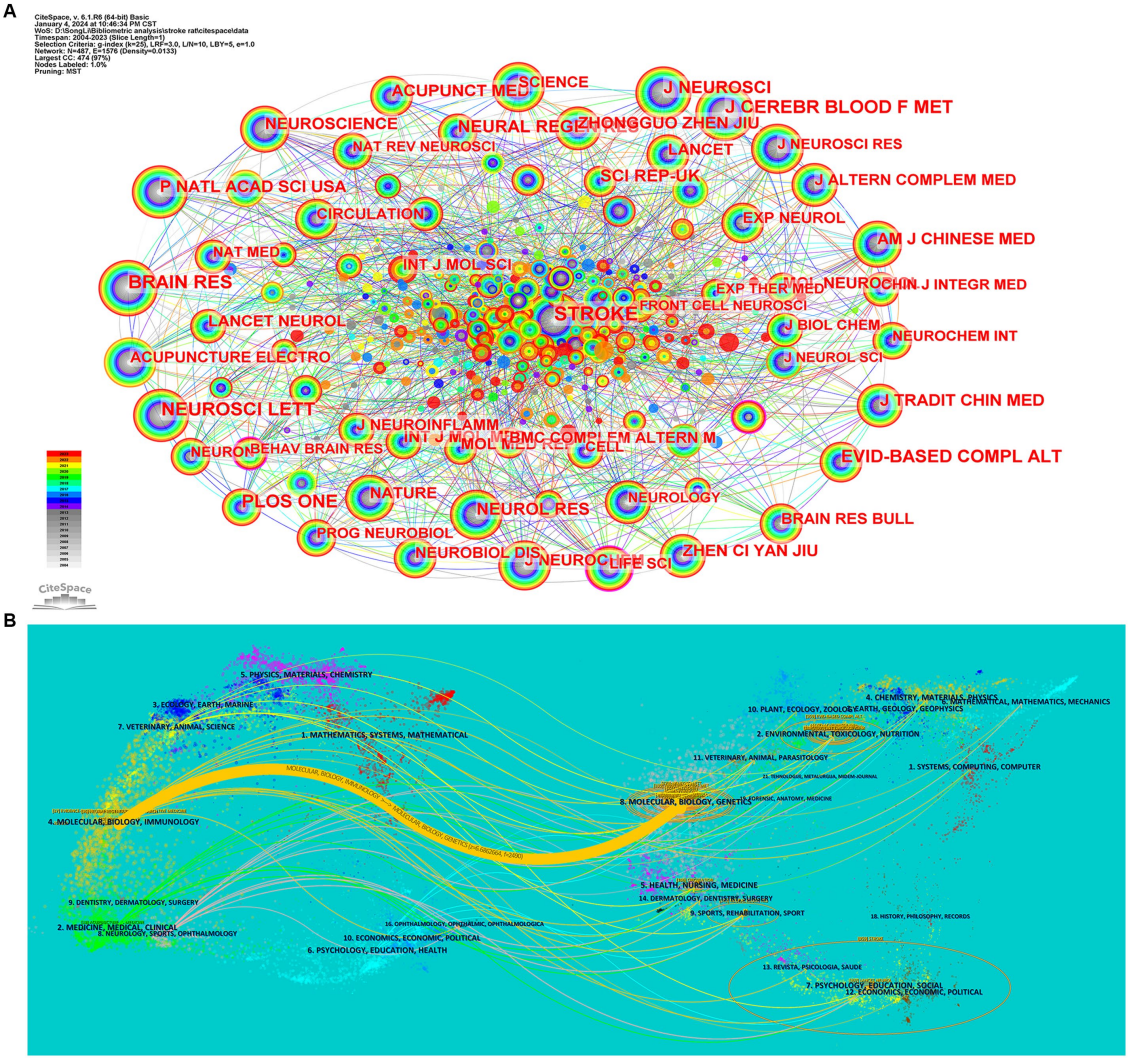
**(A)** Distribution of co-cited journals in a rat model of stroke treated with acupuncture. **(B)** The dual-map overlay of journals related to PE research.

Table 3 shows the top 10 journals in terms of co-citation frequency. The Stroke Journal was the most frequently cited (313 citations). In comparison, other journals cited less regularly (less than 200 citations), suggesting that the Stroke Journal has an irreplaceable role in the study of acupuncture in treating stroke in rat models. Figure 3A shows the network map of co-cited journals. Figure 3B shows a double map overlay of the journals, with the left and right sides corresponding to the citation map and the cited journal map, respectively. The labels represent the disciplines covered by the journals. The lines on the map start on the left, end on the right, and represent citation links. The only cited path is the orange one. MOLECULAR/BIOLOGY/IMMUNOLOGY cites the field of MOLECULAR/BIOLOGY/GENETICS (*z* = 6.6862664, *f* = 2,490).

**Table 3:** Top 10 co-cited journals for studies related to acupuncture for stroke rat models.

Ranking	Journal	Centrality	Counts
1	Stroke	0.02	313
2	Brain Res	0.06	198
3	Neurosci Lett	0.03	195
4	J Cerebr Blood F Met	0.06	183
5	Plos One	0.01	169
6	Evid-Based Compl Alt	0.02	167
7	J Neurosci	0.03	145
8	Neural Regen Res	0.04	132
9	Neurol Res	0.02	121
10	Sci Rep-Uk	0.01	121

### 2.5 Author’s and co-authorship analysis

**Figure 4:**
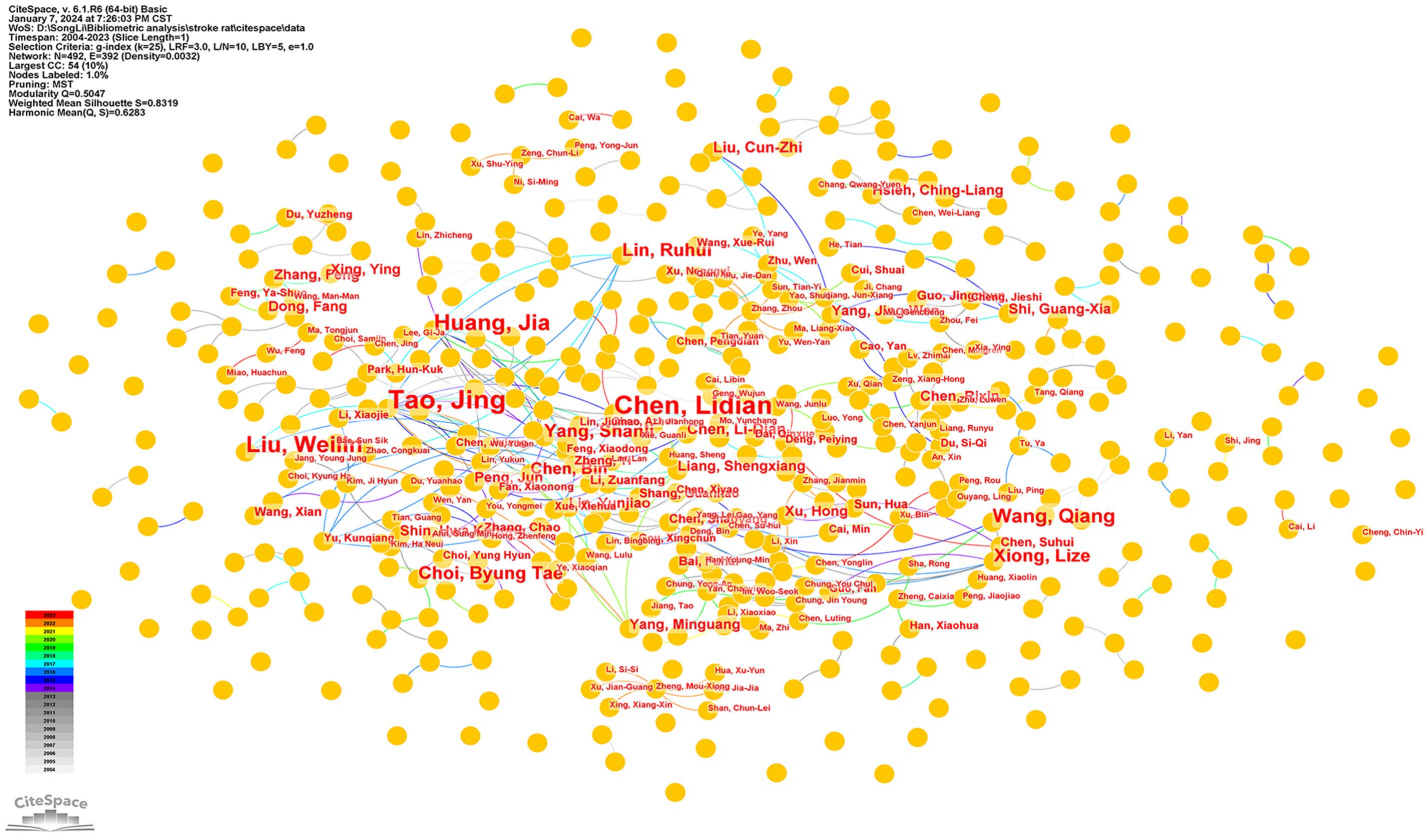
Distribution of authors in the field of acupuncture for stroke rat models research.

We visualized 492 authors for 379 publications (Figure 4). Regarding publication output, Tao, Jing, and his team are the authors who published the most papers in this field, with 33 papers. Followed by Chen, Lidian (29 papers), Liu, Weilin (19 papers), Huang, Jia (18 papers) and Wang, Qiang (12 papers). Table 4 shows the top 10 authors regarding the frequency of publications in this field. The author visualization shows how closely authors collaborate, which can provide information about influential research groups and potential collaborators (Figure 4). Among the most cited authors, excluding authors with unknown citations, we can draw the following conclusions: Longa EZ ranked first with 144 citations, followed by Wang Q (60 citations), Liu WL, Tao J (40 citations), Kim JH, Chavez LM (38 citations) and Feng XD (36 citations) (Table 4).

**Table 4:** Top 10 authors and co-cited authors of the study of acupuncture for stroke in a rat model.

Ranking	Authors	Count	Ranking	Frequency	Co-cited author
1	Tao, Jing	33	1	144	Longa EZ
2	Chen, Lidian	29	2	60	Wang Q
3	Liu, Weilin	19	3	40	Liu WL
4	Huang, Jia	18	4	40	Tao J
5	Wang, Qiang	12	5	38	Kim JH
6	Choi, Byung Tae	9	6	38	Chavez LM
7	Lin, Ruhui	9	7	36	Feng XD
8	Xiong, Lize	9	8	35	Bederson JB
9	Yang, Shanli	9	9	33	Kim YR
10	Chen, Bin	7	10	32	Li J

### 2.6 Institutional analysis

**Figure 5:**
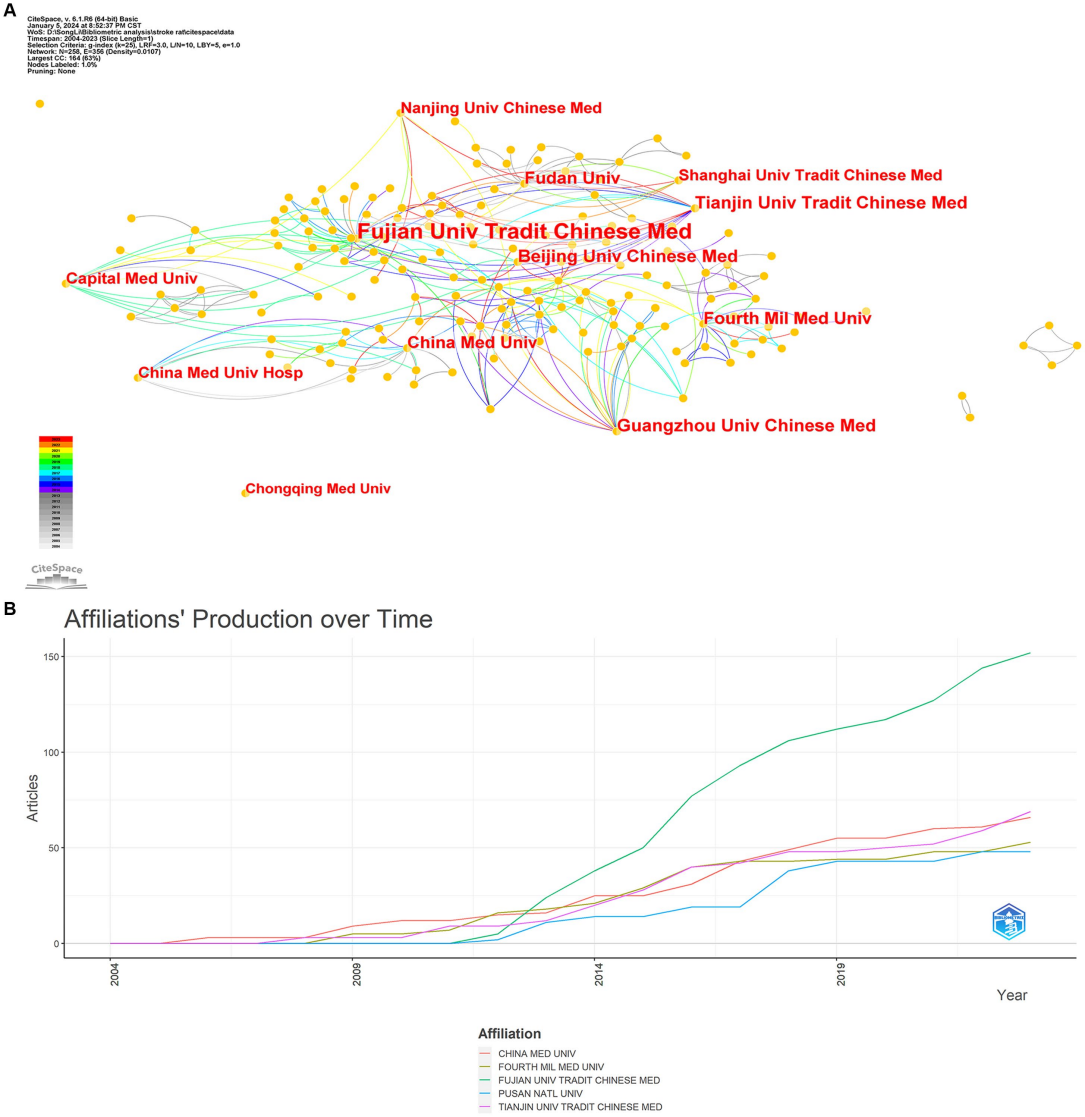
Affiliation analysis. **(A)** Network diagram of publishing institutions using CiteSpace. **(B)** Affiliation production over time depicted using bibliometrics.

This study uses CiteSpace to analyze the research on acupuncture treatment of rat stroke model. The results showed that 258 institutions participated in research in this field (Figure 5A). As shown in Figure 5A, close collaboration between institutions still needs to be improved. Figure 5B shows the 5 affiliated institutions that have been active in publishing papers recently. Table 5 shows the five most productive institutions. The institution with the highest number of publications is Fujian University of Traditional Chinese Medicine (11.35%, 43 articles), followed by Tianjin University of Chinese Medicine, Guangzhou University of Chinese Medicine, Beijing University of Chinese Medicine, Beijing University of Chinese Medicine (5.01%, 19 articles), Fudan University, Fourth Military Master’s School of Medicine (4.49%, 17 articles), China Medical University (4.22%, 16 articles) and Capital Medical University (3.69%, 14 articles). Fujian University of Traditional Chinese Medicine had the highest centrality (0.27). It shows that this institution collaborates most frequently with other institutions and could be a potential partner for future research institutions.

**Table 5:** Analysis of the number of papers from the top 5 organizations in the field of acupuncture for stroke rat models research.

Ranking	Institution	Centrality	Counts	%(of 379)
1	Fujian Univ Tradit Chinese Med	0.29	43	11.35
2	Tianjin Univ Tradit Chinese Med	0.09	19	5.01
2	Guangzhou Univ Chinese Med	0.18	19	5.01
2	Beijing Univ Chinese Med	0.11	19	5.01
3	Fudan Univ	0.18	17	4.49
3	Fourth Mil Med Univ	0.14	17	4.49
4	China Med Univ	0.09	16	4.22
5	Capital Med Univ	0.25	14	3.69

### 2.7 Keyword analysis

**Figure 6:**
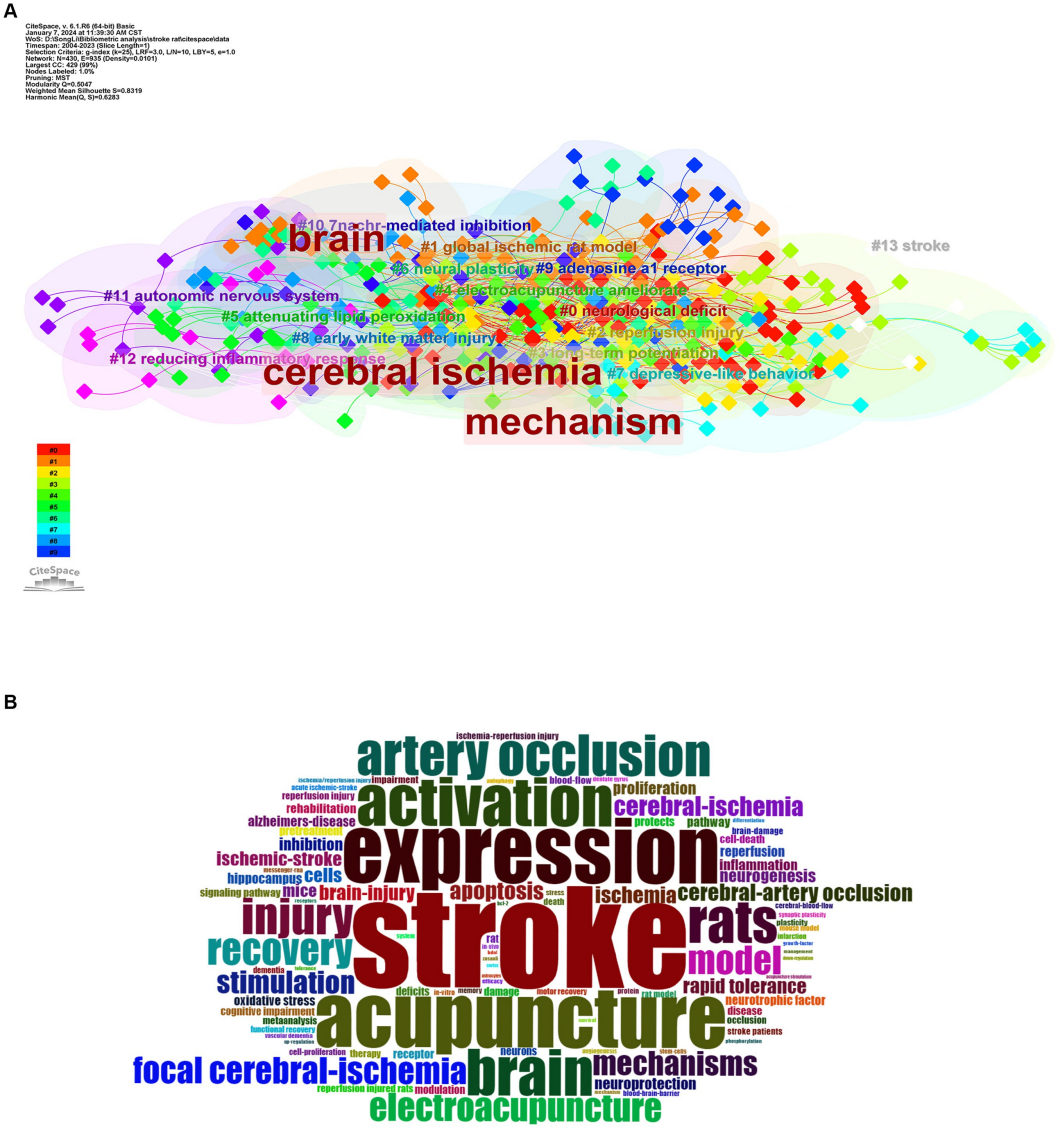
Keyword analysis. **(A)** Clustering diagram of co-occurring keywords related to acupuncture for stroke rat models. **(B)** Keyword cloud of co-occurring keywords related to acupuncture for stroke rat models.

**Figure 7:**
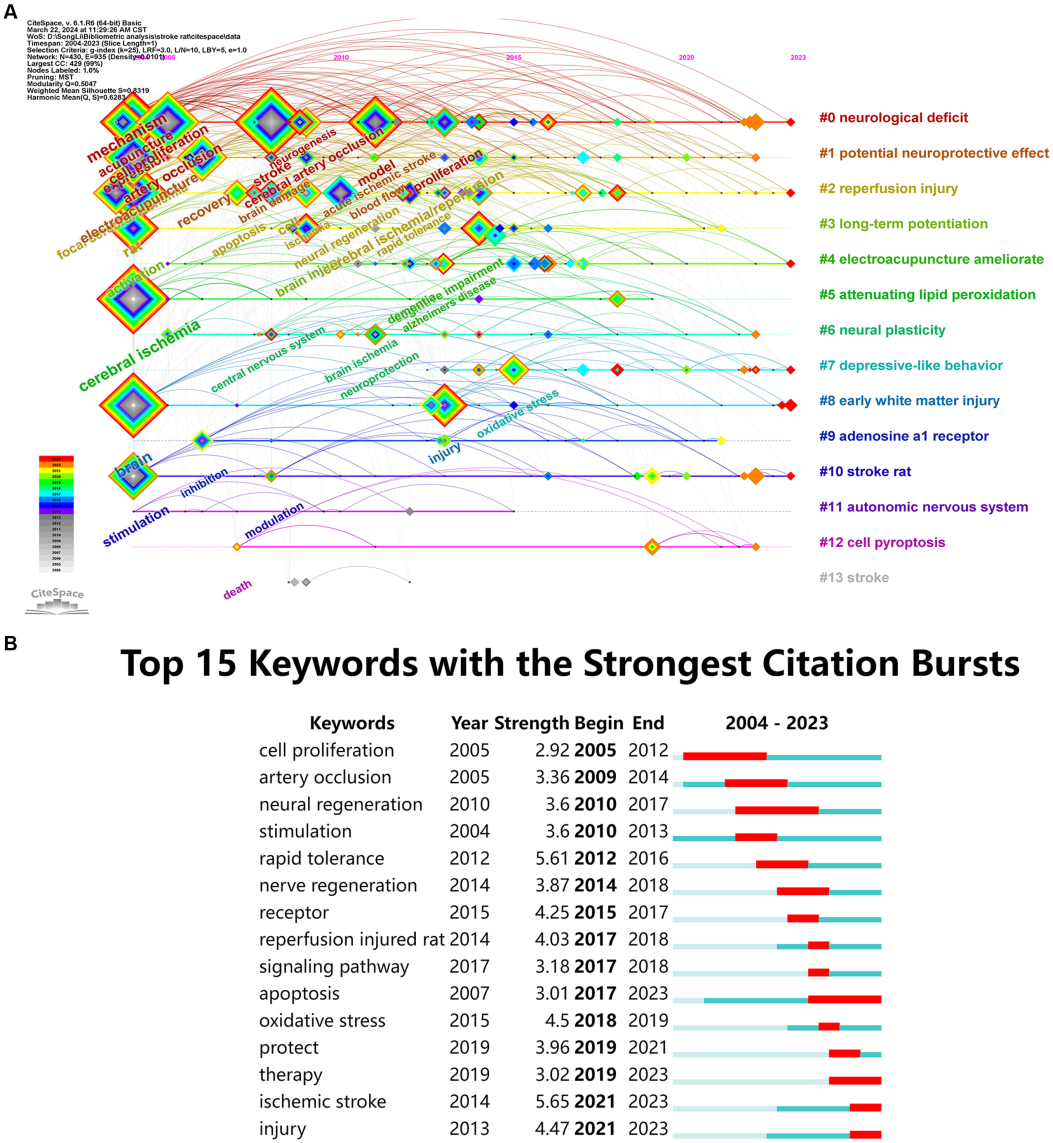
**(A)** Timeline of keywords in the field of research on acupuncture for stroke rat models. **(B)** Top 15 keywords with the strongest citation bursts.

We used CiteSpace to visualize and analyze the keywords for this area of research, including 430 nodes and 935 links for a total of 430 keywords (Figures 6A,B). Table 6 shows the 10 most commonly used co-citation keywords. The 10 most frequently used co-cited keywords are “stroke” (141), followed by “expression” (91), “acupuncture” (87), “rat” (71), “activation” (68), “cerebral ischemia” (67), “brain” (66), “artery occlusion” (66), “ischemic stroke” (56) and “injury”(46). The 10 most central keywords were “brain” (0.29), “cerebral ischemia” (0.24), “mechanism”(0.24), “recovery” (0.24), “activation” (0.24), “artery occlusion” (0.24), “stroke” (0.24), “electroacupuncture” (0.24), “acupuncture“(0.24) and “rat” (0.14). Therefore, we believe that the future research hotspots in the field of acupuncture for stroke rat models will be in the areas of “brain,” “activation,” “expression,” “mechanism,” and “recovery.” Typically, a cluster with a silhouette value >0.5 is reasonable, while a value >0.7 indicates that the cluster is convincing. The Weighted Mean Silhouette S = 0.8319 for this cluster suggests that the cluster is convincing. We analyzed the keywords with 14 clusters (Figure 6A). Explicitly including the following clusters: #0 neurological deficit, #1 global ischemic rat model, #2 reperfusion injury, #3 long-term potentiation, #4 electroacupuncture ameliorate, #5 attenuating lipid peroxidation, #6 neural plasticity, #7 depressive-like behavior, #8 early white matter injury, #9 adenosine a1 receptor, #10 7nachr-mediated inhibition, #11 autonomic nervous system, #12 reducing inflammatory response, #13 stroke. Figure 7A uses a timeline vision to show the evolutionary timeline of keywords.

**Table 6:** Top 10 co-occurring keywords in the field of acupuncture for stroke rat models research.

Ranking	Keyword	Counts	Centrality	Ranking	Keyword	Counts	Centrality
1	stroke	141	0.12	1	Brain	66	0.29
2	expression	91	0.09	2	Cerebral ischemia	67	0.24
3	acupuncture	87	0.11	3	Mechanism	40	0.21
4	rat	71	0.11	4	Recovery	37	0.17
5	activation	68	0.14	5	Activation	68	0.14
6	cerebral ischemia	67	0.24	6	Artery occlusion	66	0.13
7	brain	66	0.29	7	Stroke	141	0.12
8	artery occlusion	66	0.13	8	Electroacupuncture	36	0.12
9	ischemic stroke	56	0.01	9	Acupuncture	87	0.11
10	injury	46	0.05	10	Rat	71	0.11

A “burst word” is a keyword used more frequently in a specific period and is a popular research topic. For example, Figure 7B shows the Top 15 keywords with the strongest citation explosion. These include “arterial occlusion,” “nerve regeneration,” “stimulation,” “rapid tolerance,” “nerve regeneration,” “receptor,” “signaling pathway,” “apoptosis,” and “oxidative stress” with higher burst intensity. “Oxidative stress” is a current research hotspot and is likely to continue in the future, and this area may provide research direction for initial researchers. The primary purpose of acupuncture intervention was to “protect” and “therapy.” Among them, the most common intervention model for stroke is the perfusion injured rat. Currently, “apoptosis,” “therapy,” “ischemic stroke,” and “injury” are still hot research topics in this field and will probably continue in the future.

### 2.8 Reference co-citation analysis

In the past 20 years, articles on acupuncture treating stroke rat models cited 679 relevant documents. The top 10 cited literature are based on citation frequency (Table 7). The top 3 references in terms of citation frequency were Lina Chavez and her team’s 2017 publication in the International Journal of Molecular Sciences entitled “Mechanisms of Acupuncture Therapy in Ischemic Stroke Rehabilitation: a Literature Review of Basic Studies” with the most frequently cited article (29 citations) (Chavez et al., 2017). Followed by Xiaodong Feng and her team’s article published in Molecular Medicine Reports, “Electroacupuncture ameliorates cognitive impairment through inhibition of NF-κB-mediated neuronal cell apoptosis in cerebral ischemia–reperfusion injured rats” (27 times) (Feng et al., 2013). Finally, Ji Hyun Kim and his team published in the journal PLOS ONE, “Electroacupuncture Acutely Improves Cerebral Blood Flow and Attenuates Moderate Ischemic Injury via an Endothelial Mechanism in Mice” (23 citations) (Kim et al., 2013).

**Table 7:** Top 10 articles with citation frequency in the study of acupuncture for the treatment of a rat model of stroke.

Ranking	References	Journal	IF (2023)	First author	Publication time	Total citations
1	Mechanisms of Acupuncture Therapy in Ischemic Stroke Rehabilitation: A Literature Review of Basic Studies	International Journal of Molecular Sciences	5.6	Lina Chavez	2017	29
2	Electroacupuncture ameliorates cognitive impairment through inhibition of NF-κB-mediated neuronal cell apoptosis in cerebral ischemia–reperfusion injured rats	Molecular Medicine Reports	3.4	Xiaodong Feng	2013	27
3	Electroacupuncture Acutely Improves Cerebral Blood Flow and Attenuates Moderate Ischemic Injury via an Endothelial Mechanism in Mice	PLOS ONE	3.7	Ji Hyun Kim	2013	23
4	Electroacupuncture exerts anti-inflammatory effects in cerebral ischemia–reperfusion-injured rats via suppression of the TLR4/NF-κB pathway	International Journal of Molecular Medicine	5.4	Lan Lan	2013	22
5	Electroacupuncture Promotes Post-Stroke Functional Recovery via Enhancing Endogenous Neurogenesis in Mouse Focal Cerebral Ischemia	PLOS ONE	3.7	Yu Ri Kim	2014	19
6	Activation of Epsilon Protein Kinase C-Mediated Anti-Apoptosis Is Involved in Rapid Tolerance Induced by Electroacupuncture Pretreatment Through Cannabinoid Receptor Type 1	Stroke	8.3	Qiang Wang	2011	17
7	Pretreatment With Electroacupuncture Induces Rapid Tolerance to Focal Cerebral Ischemia Through Regulation of Endocannabinoid System	Stroke	8.3	Qiang Wang	2009	16
8	Electro-acupuncture at LI11 and ST36 acupoints exerts neuroprotective effects via reactive astrocyte proliferation after ischemia and reperfusion injury in rats	Brain Research Bulletin	3.8	Jing Tao	2016	15
9	Electroacupuncture at the Quchi and Zusanli acupoints exerts a neuroprotective role in cerebral ischemia–reperfusion injured rats via activation of the PI3K/Akt pathway	International Journal of Molecular Medicine	5.4	Azhen Chen	2012	15
10	Electroacupuncture pretreatment attenuates cerebral ischemic injury through α7 nicotinic acetylcholine receptor-mediated inhibition of high-mobility group box 1 release in rats	Journal of Neuroinflammation	9.3	Qiang Wang	2012	14

Among the top 10 citations regarding citation frequency, the other 7 dealt with interventions combining classical acupuncture with modern technology (electricity). The topics covered areas such as the inhibition of the TLR4/NF-κB pathway (Lan et al., 2013), increased endogenous neurogenesis (Kim et al., 2014), apoptosis (Wang et al., 2011), induction of rapid tolerance to focal cerebral ischemia (Wang et al., 2009, 2011), the proliferation of reactive astrocytes (Tao et al., 2016), neuroprotective effect (Chen et al., 2012) and inhibition of the release of the α7 nicotinic acetylcholine receptor (Wang et al., 2012). Mechanisms of the rat models of acupuncture treatment for stroke illustrated from different perspectives, such as the inflammatory response (Lan et al., 2013), apoptosis (Wang et al., 2011), tolerance of local cerebral ischemic tissues(Wang et al., 2009), proliferation of reactive astrocytes (Tao et al., 2016), and receptors (Wang et al., 2012). The impact factors of the top 10 cited journals regarding citation frequency in 2023 range from 3.4 to 9.3. It shows that the quality of cited articles in this field is still low.

**Figure 8:**
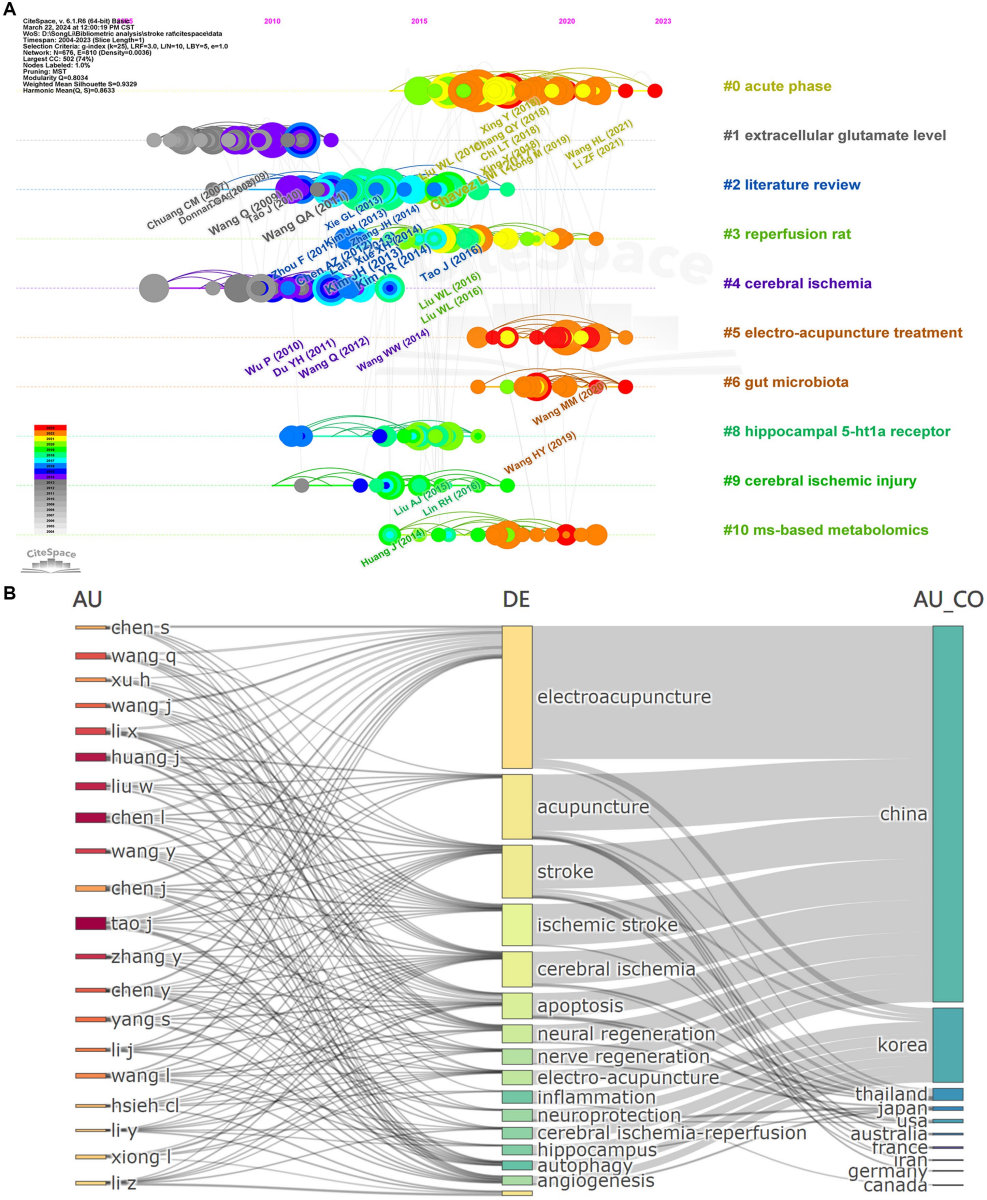
**(A)** Timeline chart of references in the field of study related to acupuncture for the treatment of a rat model of stroke. **(B)** Analysis of the relationship between authors, keywords, and countries.

In addition, we used CiteSpace to visually analyze the timeline view of literature related to acupuncture treatment of stroke rat model (Figure 8A). Its parameters are set to the log-likelihood ratio (LLR) to identify hotspot distributions from 10 clustered literature. Clusters with warmer colors indicate the most recent research, and larger nodes indicate more publications, suggesting that the clustered problem is a hotspot in this field. Thus, cluster #0 (acute phase), cluster #2 (literature review), cluster #3 (reperfusion rat), cluster #4 (cerebral ischemia), cluster #5 (electro-acupuncture treatment), cluster #6 (gut microbiota), cluster #8 (hippocampal 5-hl1a receptor), cluster #9 (cerebral ischemic injury), and cluster #10 (ms-based metabolomics) indicate the research hotspots in recent years for acupuncture treatment of stroke rat models.

### 2.9 Relationship between authors, keywords, countries

We applied RStudio to produce a Three-Field Plot (TFP) to demonstrate the relationship between authors, keywords, and countries (Figure 8B). The size of the rectangles represents the number of publications, while the connecting lines depict the correlation between academic strengths, with more links indicating a more significant number of studies. Tao J demonstrated the most comprehensive range of studies for the authors involved in this field and was significantly associated with the keywords electroacupuncture, stroke, apoptosis, autophagy, hippocampus, and acupuncture.

In terms of countries, Chinese authors have made an irreplaceable contribution through many publications exploring various aspects of this field. Interestingly, although the number of articles published by countries such as Korea is low, the main areas of research include “electroacupuncture,” “acupuncture,” “apoptosis,” “Stroke,” “neuroprotection,” and other related areas.

## 3 Discussion

### 3.1 General information

Among the articles published in the past 20 years, this study included 379 papers, and the number of published papers and citations has been increasing rapidly year by year. This result suggests that studies based on a rat model of acupuncture for the treatment of stroke disease have long-term appeal for researchers. Among the top 10 contributing countries/regions, the People’s Republic of China accounts for 89.18% of published papers. The research institution also has representatives from Fujian University of Traditional Chinese Medicine, reflecting the high acceptance of acupuncture in China. In addition, since acupuncture treatment originated in ancient China, it is more recognized and accepted in China, and its theoretical inheritance is heavily based on the experience of doctors. With the further integration of traditional and modern medicine, more and more researchers are gradually shifting from studying the efficacy of acupuncture to exploring the mechanism of acupuncture. These reasons may explain why the literature included in this study comes more from the research results of Chinese scholars. Tao, Jing, and their team topped the list with 33 papers regarding author distribution. According to Price’s law (Zhou D. et al., 2022; Zhou R. et al., 2022), the core authors’ minimum number of publications is N = 0.749 $\sqrt{M\;\max}$ (M max is the most prolific author’s publication), calculated as N ≈ 4.3. Regarding the number of publications, 35 authors have published more than 4 articles, accounting for 7.11% (< 50%) of the total publications; this indicates that a core group of authors has yet to be formed in this research area. Currently, scholars studying acupuncture for treating rat models of stroke are mainly concentrated in Asia. Researchers must strengthen cooperation and communication between scholars from different countries to further explore the effective mechanism of acupuncture treatment.

Scholarly research should be based on established theories or evidence often articulated in cited references (Li Q. et al., 2023). The most cited reference was the Literature Review by Lina Chavez (IF = 5.6) (Chavez et al., 2017). It was mentioned 29 times out of 379 articles (70 citations). This Literature Review suggests that the beneficial effects of acupuncture on ischemic stroke rehabilitation involve five main distinct mechanisms: (1) promotion of neurogenesis and cell proliferation in the central nervous system (CNS); (2) modulation of cerebral blood flow in the ischemic region; (3) anti-apoptosis in the ischemic region; (4) modulation of neurochemicals; and (5) improvement of impaired post-stroke long-term potentiation (LTP) and memory. The most frequently used acupoints in basic studies include Baihui (GV20), Zusanli (ST36), Quchi (LI11), Shuigou (GV26), Dazhui (GV14), and Hegu (LI4). The second most cited article is Xiaodong Feng’s 2013 article in Molecular Medicine Reports (IF = 3.4) (Feng et al., 2013), “Electroacupuncture ameliorates cognitive impairment Electroacupuncture ameliorates cognitive impairment through inhibition of NF-κB-mediated neuronal cell apoptosis in cerebral ischemia–reperfusion injured rats” published in Molecular Medicine Reports (IF = 3.4) in 2013. It was cited 27 times in this field and 37 times in total. This study suggests that electroacupuncture stimulation of Baihui point (DU20) and Shenting point (DU24) may inhibit NF-κB-mediated neuronal cell apoptosis by significantly down-regulating the expression of pro-apoptotic Bax and Fas, two critical downstream target genes of the NF-κB pathway, and thus achieve therapeutic effects on post-stroke cognitive impairment.

Among the 10 most frequently cited articles in the selected field, their impact factors 2023 range from 3.4 to 9.3. Chen, Lidian and his team used electroacupuncture to intervene in the Quchi and Zusanli acupoints of the affected side of the adult male Sprague–Dawley rat ischemia–reperfusion model and found that electroacupuncture can increase the proliferation of reactive astrocytes, activate PI 3 K/Akt, and inhibit TLR4/NF-κB signaling pathway, thereby regulating inflammatory response and apoptosis to exert neuroprotective effects (Chen et al., 2012; Lan et al., 2013; Tao et al., 2016). However, a blank pair and sham acupuncture group were not established. The test results cannot be determined to be caused by acupuncture of Quchi and Zusanli acupoints. It is unclear whether acupuncture of non-acupoints and Quchi and Zusanli acupoints on the contralateral side will produce the same results. Therefore, the study has certain flaws that reduce the credibility of its evidence. Similar problems also exist in other papers selected in this study. In summary, the quality of research in this field should be further optimized and improved.

### 3.2 Hot research topics

This study aims to investigate the research trends and hot spots in acupuncture in the rat stroke models. We used CiteSpace and R Studio to visualize and analyze the keywords, discovering that “stroke,” “acupuncture,” and “rat” should be the most frequently used keywords. There is a growing emphasis in both clinical treatment and research on understanding the mechanism of action in the stroke recovery period, as well as brain cell damage and changes in brain functional areas. Therefore, we believe that topics such as “brain,” “activation,” “expression,” “mechanism,” and “recovery” may be hot spots for future research.

In the area of “burst word” analysis. We found that research in this field is currently in a rapid development stage, predominantly focusing on arterial occlusion, nerve regeneration, stimulation, rapid tolerance, receptors, signaling pathways, apoptosis, and oxidative stress. In terms of studying stroke types, research in this field focuses more on exploring the impact of acupuncture treatment on atherosclerotic cerebral infarction. Most of the included studies used middle cerebral artery occlusion (MCAO) animal models prepared by the suture-occluded method to simulate the ischemia–reperfusion characteristics of clinical cerebral infarction. The reason may be that the incidence of cerebral infarction accounts for 70–80% of the incidence of stroke, and atherosclerosis is the most common cause of cerebral infarction (Kang et al., 2010; Wu et al., 2021). During the early stage of ischemic stroke, cerebral ischemia significantly reduces the supply of nutrients such as glucose and oxygen, leading to inadequate or exhausted energy reserves in nerve cells, culminating in cell edema, necrosis, demyelination, and axonal demyelination (Yuan et al., 2021). Studies have confirmed that electroacupuncture can regulate the mTOR signaling pathway to induce neuroprotection and neuroplasticity, thereby promoting axon regeneration in the contralateral cortex and corticospinal tract, activating neuroplasticity, and achieving the purpose of neurological recovery (Zheng et al., 2016; Chen et al., 2020; Zhang S. H. et al., 2022; Zhang Y. et al., 2022). Neural regeneration, angiogenesis, and synaptic regeneration are crucial for autonomic plasticity in the brain, potentially elucidating the positive clinical effects in patients with cerebral infarction. Therefore, future scholars may be interested in Neural regeneration. Scalp acupuncture treatment is closely related to the functional positioning of the cerebral cortex. Acupuncture stimulates the corresponding functional areas to treat diseases (Liu et al., 2019; Peng et al., 2023). PKC, a family of widely expressed serine/threonine kinases, mediates rapid and delayed preconditioning within the central nervous system. Epsilon protein kinase C (εPKC) is critical for inducing ischemic tolerance in various preconditioning models. Studies have confirmed that electroacupuncture preconditioning can activate endogenous εPKC-mediated anti-apoptosis mechanisms, prevent ischemic damage after focal cerebral ischemia through cannabinoid receptor type 1, and enhance the tolerance of brain tissue to ischemia and hypoxia (Wang et al., 2009, 2011). Receptors are a class of molecules that can transmit extracellular signals and produce specific effects within cells. Studies have confirmed that regulating gamma-aminobutyric acid (GABA) and glycogen synthase kinase 3 (GSK-3) has neuroprotective effects. Acupuncture can protect nerve and reverse motor function by regulating the expression of GABA receptors in MCAO rats. In addition, electroacupuncture preconditioning can prevent cerebral ischemia/reperfusion injury through cannabinoid CB1 receptor (CB1R)-mediated phosphorylation of glycogen synthase kinase-3β (GSK-3β) (Wei et al., 2014; Xu et al., 2015; Li et al., 2022). Following brain injury, the brain produces abnormal signals, which are transmitted through signaling pathways and reach cells to exert effects. The PI3K/Akt, Wnt/β-catenin, and NF-κB signaling pathways play key roles in cell growth, proliferation, differentiation, movement, survival, and intracellular trafficking. Research has confirmed that electroacupuncture can promote the proliferation of peripheral neural progenitor cells (NPCs) in the regions surrounding the cortical infarction after stroke by upregulating the Wnt/β-catenin signaling pathway, thereby exerting a therapeutic effect on cerebral ischemia. At the same time, electroacupuncture can also inhibit the NF-κB signaling pathway by acting on the deubiquitinase (OTULIN) target to relieve brain damage and glial cell activation induced by an acute ischemic stroke. Furthermore, studies have confirmed that electroacupuncture can exert neuroprotective functions by activating the PI3K/Akt pathway in patients with ischemic stroke (Chen et al., 2012¸ 2015; Xu et al., 2021). Apoptosis is a common pathological change in cerebral ischemia and hypoxia. Modulating apoptotic signaling pathways helps reduce neuronal cell death after focal or global ischemic stroke. Cysteinyl aspartate-specific proteinase (caspase), a group of proteases in the cytoplasm, plays an irreplaceable role in apoptosis and inflammatory responses. The study found that electroacupuncture treatment can significantly reduce ischemic stroke-induced caspase-3 and the up-regulation of caspase-3, thereby reversing the down-regulation of the b-lymphoma-2 gene (Bcl-2) and reducing apoptosis. The intervention reduces patient mortality and induces neuroprotective effects (Chuang et al., 2013; Xing et al., 2018a,b). During cerebral ischemia and ischemia/reperfusion, brain cells produce excess reactive oxygen species (ROS), leading to cytotoxic effects through oxidative damage to lipids, proteins, and nucleic acids. This process can also lead to neuronal apoptosis, activation of inflammatory signaling pathways, and damage to the blood–brain barrier (BBB), thereby promoting neurodegeneration and cell death (Su et al., 2020). A large number of animal studies have shown that acupuncture/electroacupuncture can reduce oxidative stress after stroke via different pathways, such as regulating iron overload, inhibiting NF-κB-p53 activation, reducing cytotoxicity, and alleviating neurodegeneration and cell death (Yang et al., 2019; Liang et al., 2021). In short, the analysis of “burst word” indicates that future researchers may pay more attention to research on arterial occlusion, nerve regeneration, stimulation, rapid tolerance, receptors, signaling pathways, apoptosis, and oxidative stress. Continued exploration into the potential mechanisms of acupuncture in stroke will be a hot topic for future research.

In terms of treatment methods, we found that electroacupuncture was mainly used, with a higher centrality (0.12) than traditional acupuncture (0.11). It serves as a combination of traditional acupuncture and modern technology. The current research on this treatment method is more prominent in the field of the rat models of acupuncture treatment for stroke, which will also likely be a future research hotspot in this field to continue to develop. Electroacupuncture has proven effective for stroke patients (Liu et al., 2015; Cai et al., 2017). Electroacupuncture allows for more precise control of acupuncture parameters, such as frequency and stimulation than conventional acupuncture. However, the relationship between the efficacy of electroacupuncture in treating stroke and the amount of stimulation needs more experiments to verify, and this may also become a hot trend in future clinical and basic research in this field.

In analyzing co-citation documents on acupuncture treatment for rat stroke models, we analyzed the 10 most frequently cited documents. The two topics of inflammatory response and reactive astrocyte proliferation are essential in this field, which is in vigorous development. Studies have indicated that electroacupuncture can inhibit the expression of several important proteins and molecules, such as nucleotide-binding oligomerization domain-like receptor protein 3 (NLRP3), pro-caspase-1, cleaved caspase-1 p20, and pro-interleukin-1β (IL-1β), tumor necrosis factor α (TNF-α), cleaved IL-1β and GSDMD. This inhibition promotes angiogenesis and inhibits inflammation, promoting the survival of transplanted mobile genetic element neural progenitor cells and reducing cerebral ischemia–reperfusion. After caspase 1-mediated pyroptosis of neurons and inflammatory response to exert neuroprotective effects (Li J. et al., 2021; Cai et al., 2022). Reactive astrocytes can protect surrounding healthy brain tissue from exposure to cellular debris and cytotoxic elements in the infarcted area or combat cerebral ischemia-induced edema by eliminating reactive oxygen species (ROS) and glutamate. In addition, it can also exert neuroprotective effects by secreting neurotrophic factors (de Pablo et al., 2013). In addition, electroacupuncture also promotes the proliferation of reactive astrocytes and exerts neuroprotective effects (Tao et al., 2016). We believe that inflammation and the proliferation of reactive astrocytes are topics that future researchers will likely continue exploring. Analysis of 10 clusters of 679 total citations reveals that, beyond the above research topics, existing research should also focus on the following aspects: #1: the timing of acupuncture intervention in stroke treatment remains controversial: whether interventions should occur in the acute phase of stroke and whether early intervention improves outcomes. #2: In recent years, studies have confirmed that intestinal commensal bacteria can regulate ischemic stroke damage and that there is a two-way communication channel between the brain and intestines - the “microbiome-gut-brain axis” (MGBA) (Agirman et al., 2021). The gut-brain axis may directly or indirectly innervate the enteric nervous system through nerve fibers, affecting the level of intestinal mucosal tight junctions and maintaining the integrity of the intestinal mucosal barrier and intestinal microbiota (Crapser et al., 2016). After cerebral ischemia, the gut microbiota further damages the immune system and aggravates intestinal and brain damage (Wang et al., 2022). Electroacupuncture has been shown to regulate intestinal immunity through MGBA, reducing brain and colon damage after cerebral ischemia and positively affecting the outcome of ischemic stroke and preventing its occurrence (Li et al., 2022; Zhang et al., 2023). #3: Cognitive dysfunctions such as memory, attention, judgment, executive functioning, and calculation are common complications for patients in the middle, which leads to a reduced quality of life (Liu et al., 2014). The hippocampus is a primary target of brainstem dopaminergic neurons whose postsynaptic serotonin (1A) receptors (5-HT 1A receptors) mediate fear, anxiety, and learning. Previous studies have shown that electroacupuncture can directly phosphorylate NMDA receptors through 5-HT 1A receptor-mediated protein kinase A (PKA) to improve the recovery of cognitive function after ischemic stroke (Wang Z. et al., 2020). #4: Metabolomics studies the composition and content changes of small molecule metabolites expressed by organisms at a specific time and space, thereby finding the relative relationship between metabolites and phenotypic changes. Using 16S rRNA gene sequencing technology and metabolomics based on Liquid Chromatography-Mass Spectroscopy (LC/MS), studies have validated the synergistic effects of combining acupuncture with NaoMaiTong in treating ischemic stroke (Xian et al., 2022). Further elaborating on the mechanism of action of acupuncture by studying the metabolomic changes in patients with stroke treated with acupuncture may be another research trend for future researchers. Of course, these themes will likely continue to evolve in the future. In addition, their studies mainly selected the reperfusion stroke model for relevant studies. The studies, themes on exosomes (Zhang S. et al., 2020), Ferroptosis (Li G. et al., 2021; Wu et al., 2023), microglia (Liu et al., 2016; Zou et al., 2022) and mitochondrial autophagy (Wang et al., 2019) may be the hotspots for future research.

### 3.3 Limitations

Our study has some limitations. (1) We only included studies in the WOS database, which showed that acupuncture is widely used in China. (2) The authors may have had a subjective view of the reference citations. (3) This study only examined 379 articles in the literature related to acupuncture for treating stroke in the rat models over the past 20 years, which may have the bias of incomplete inclusion of literature.

## 4 Conclusion

This bibliometric study sheds light on overall global publishing trends and evolving research trends over the past 20 years. Finally, potential collaborators, research institutions, research hotspots, future research trends, and the relationship between authors, keywords, and countries in this field are analyzed to inform beginners of the research direction. The analysis shows a rapid and steady increase in research for the rat models of acupuncture for stroke. In addition, the leading journals published in this field are Evidence-Based Complementary and Alternative Medicine. Besides, although The People’s Republic of China has the most significant output of environmental impact research, there must be more international collaboration. The overall quality of research is increasing as the research methods and techniques improve. However, with the limitation of understanding things, the mechanism of action of acupuncture in treating stroke disease still needs to be made public. The mechanism of action of acupuncture in treating stroke needs further study.

## Author contributions

SL: Conceptualization, Data curation, Formal analysis, Funding acquisition, Investigation, Methodology, Project administration, Resources, Software, Supervision, Validation, Visualization, Writing – original draft, Writing – review & editing. ZH: Conceptualization, Investigation, Methodology, Supervision, Visualization, Writing – original draft, Writing – review & editing. TZ: Investigation, Software, Supervision, Validation, Writing – original draft, Writing – review & editing. AD: Methodology, Software, Supervision, Visualization, Writing – original draft, Writing – review & editing. XC: Investigation, Methodology, Software, Validation, Writing – original draft, Writing – review & editing. XY: Investigation, Methodology, Software, Supervision, Validation, Visualization, Writing – original draft, Writing – review & editing. LZ: Methodology, Software, Supervision, Writing – original draft, Writing – review & editing. YC: Methodology, Software, Supervision, Validation, Writing – original draft, Writing – review & editing. JS: Software, Supervision, Validation, Visualization, Writing – original draft, Writing – review & editing, Conceptualization, Data curation, Formal analysis, Funding acquisition, Investigation, Methodology, Project administration, Resources.

## Footnotes

^1^ https://www.ncbi.nlm.nih.gov/mesh
